# Infants’ Learning of Phonological Status

**DOI:** 10.3389/fpsyg.2012.00448

**Published:** 2012-11-02

**Authors:** Amanda Seidl, Alejandrina Cristia

**Affiliations:** ^1^Purdue UniversityWest Lafayette, IN, USA; ^2^Neurobiology of Language, Max Planck Institute for PsycholinguisticsNijmegen, Netherlands

**Keywords:** infants, perception, phonology, phonemes, speech

## Abstract

There is a substantial literature describing how infants become more sensitive to differences between native phonemes (sounds that are both present and meaningful in the input) and less sensitive to differences between non-native phonemes (sounds that are neither present nor meaningful in the input) over the course of development. Here, we review an emergent strand of literature that gives a more nuanced notion of the problem of sound category learning. This research documents infants’ discovery of *phonological status*, signaled by a decrease in sensitivity to sounds that map onto the same phonemic category vs. different phonemic categories. The former phones are present in the input, but their difference does not cue meaning distinctions because they are tied to one and the same phoneme. For example, the diphthong *I* in *I’m* should map to the same underlying category as the diphthong in *I’d*, despite the fact that the first vowel is nasal and the second oral. Because such pairs of sounds are processed differently than those than map onto different phonemes by adult speakers, the learner has to come to treat them differently as well. Interestingly, there is some evidence that infants’ sensitivity to dimensions that are allophonic in the ambient language declines as early as 11 months. We lay out behavioral research, corpora analyses, and computational work which sheds light on how infants achieve this feat at such a young age. Collectively, this work suggests that the computation of complementary distribution and the calculation of phonetic similarity operate in concert to guide infants toward a functional interpretation of sounds that are present in the input, yet not lexically contrastive. In addition to reviewing this literature, we discuss broader implications for other fundamental theoretical and empirical questions.

## Introduction

There is a large literature on how infants become more sensitive to differences between phones that map onto different native phonemes (sounds that are both present and meaningful in the input) and less sensitive to differences between phones that map onto different non-native phonemes (sounds that are neither present nor meaningful in the input) as they mature. This literature shows that infants begin to zero-in on the phonemes present in their native language sometime between 4 and 12 months of age (Werker and Tees, 1984; Polka and Werker, 1994). However, categorizing sounds as either present in, as opposed to absent from, the input is only one step in language acquisition. Certainly this helps the infant to focus on her specific language’s properties and ignore other language’s properties, and this ability to build language-specific phonetics may even be fundamental in building a lexicon (Kuhl et al., 2008). However, the child must also learn to categorize sounds which are present, but not meaningful in the input language. This task is likely to recruit the same mechanisms as the native/non-native task. Specifically, in every language, there are sounds that are present in the input but do not map onto different native phonemes, since their different forms do not cue meaning distinctions and the child must learn to map these to a unified phonemic representation. For example, the diphthong *I* in *I’m* should map to the same underlying category as the diphthong in *I’d*, despite the fact that one vowel is nasal and the other oral. For ease of expression and reading, we will use the shorthand of “allophones” for phones that map onto the same phonemic category, and “phonemes” for phones that map onto different phonemic categories^1^. In this paper we summarize evidence on the acquisition of allophones to answer two key questions: When and how does the learner determine whether two sounds are allophones or phonemes in the target language?

Before turning to the evidence on the acquisition of allophones and the mechanisms underlying their acquisition, it is important to discuss both how allophones and phonemes are defined within the linguistic, descriptive literature (Section “What are allophones”); and how they are processed by individuals with a fully developed grammar according to the psycholinguistic literature (Section “The end state”). We then review an emergent strand of literature that documents when infants begin to apply a differential processing of allophones and phonemes (Section “Infants’ processing of allophones”) and how they might have learned to make such a distinction between allophones and phonemes (Section “Mechanisms for learning allophones”). The final section (“Implications”) discusses how research on infants’ learning of phonological status can inform, and be informed by, other areas of investigation. Throughout this article, we identify areas where answers are still lacking. We hope that this review serves as a springboard for such work and helps to point to clear areas out of which this future work can grow.

## What are Allophones?

There are two classical cases of allophony, which are commonly discussed in introductory linguistics courses (Trubetzkoy, 1939/1969; Kenstowicz, 1994). The first involves “sounds in complementary distribution.” Two sounds are in complementary distribution if the sound which should be used is completely predictable from the context; put differently, the contexts in which each sound can occur are completely non-overlapping. For example, in most varieties of American English, dark /l/ occurs syllable-finally (“ball”), whereas light /l/ occurs in all other positions (“lab”). Notice that no two words in American English differ only on whether they have a light or dark /l/. In other words, sounds in complementary distribution do not cue meaning distinctions. Finally, a third criterion for allophony in this case is that the two sounds must be somehow acoustically related, such that they may be interpreted as the “same” sound, on some abstract level. For instance, although /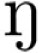/ and /h/ are in complementary distribution in English (the former occurs only in syllable codas, the latter only in syllable onsets), phonologists would not want to posit that they are allophones since they are highly acoustically distinct (Bazell, 1954).

The second classical case of allophony relates to sounds in “free variation.” In this case, speakers can produce two or more different sounds in the exact same environment (e.g., ri[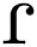]er versus ri[d]er in American English); however, these differences are not lexically relevant. Much work debates the name “free,” since in many such cases the variant which is selected appears to be explained, to a considerable extent, by a number of structural, sociolinguistic, and idiolectal variables (e.g., Fischer, 1958). Nonetheless, it remains the case that two sounds which can be thus exchanged without semantic changes can be viewed as allophones. The traditional way of establishing whether two sounds are in free variation is by carrying out a minimal pair test. Minimal pairs are two word forms that differ in only one sound; if this sound swap results in meaning change or loss, then the two sounds are phonemes, but if it does not, they are allophones in free variation.

In phonology, as in life, things can sometimes get more complicated, and for the definition of allophony this is true in a number of ways. To begin with, there are cases of complementary distribution and free variation that are true in certain phonological and lexical contexts, but not others. For example, one could state that voiceless unaspirated and voiceless aspirated stops are in complementary distribution in American English, with the former occurring e.g., after /s/ (as in “stop”) and the latter e.g., in the onset of monosyllables (as in “top”). However, voiceless unaspirated stops are minimally different from the surface realizations of voiced stops in syllable-initial position when following a word ending in /s/, to such an extent that one-year-olds fail to discriminate them (Pegg and Werker, 1997)^2^.

Moreover, sometimes two pronunciations are possible without meaning change in some structural positions (e.g., [i]conomy vs. [ə]conomy) but not in others (e.g., wom[ɪ]n vs. wom[ə]n; though perhaps a clearer example is tense and lax vowels, both of which can occur in closed syllables, but only tense vowels occur in open syllables). Additionally, some sounds would fit the definition of phonemes, but may be present in only a handful of loanwords (such as a pronunciation of the composer Bach as Ba[x] versus Ba[k]; Halle, 1964); whereas for others there may be no minimal pairs, even though the linguist’s intuition indicates that two sounds are contrastive because they are both active (used in a phonological constraint/rule) and prominent (involved in some type of phonological, morphological, or even long distance effect; Clements, 2001)^3^. Scobbie et al. (1999) and Scobbie and Stewart-Smith (2008), among others, have discussed extensively another ambiguous case from Scottish Standard English, where some vowels have long and short variants that are contextually determined, yet for which some minimal pairs, with specific morpholexical characteristics, can nevertheless be found. This is the case for long and short variants of /ai/, which contrast minimally in “side” and “sighed,” with the long version being found in morphologically complex items. In spite of the existence of such minimal pairs, the two sounds are in free variation across speakers in some lexical items, such as “crisis.”

In view of such cases both within and across languages, Pierrehumbert (2003) proposes to do away with the distinction between phonemes and allophones and instead attempt an explanation of learners’ acquisition of positional allophones, defined as clusters of tokens in acoustic space. A comparable proposal was made in Ladd (2006), who goes further by pointing out that allophones are sometimes very meaningful sociolinguistically, and are thus highly perceptually salient to native speakers. Scobbie and Stewart-Smith (2008) argue, instead, that while the concepts of allophones and phonemes may be useful end points, a continuum could exist between allophones and phonemes, and propose that speakers/listeners’ grammars could well be fuzzy. More recently, Hall (2009) makes specific proposals as to how to predict perceptibility from gradient versions of an allophony/phonemicness scale. Clearly the limitations of the classical definitions of allophones and phonemes are not new (see e.g., Pike, 1947), but they are just now beginning to gain a unique combination of linguistic *and* psycholinguistic attention as it becomes increasingly clear that such phenomena are not marginal, and that such gradience is relevant to both language learning and processing. Indeed, a look through Table 1 reveals a window into the scope of this gradience. While it is not the aim of this paper to debate phonological theory, nor to enumerate cases along this continuum, we keep the question of gradience in mind when considering how infant learners may approach the phonological system, and what types of allophones versus phonemes (i.e., at what point of the continuum) have been studied in previous experimental work. With this enriched view of allophony, we now turn to adults’ perception of these two (or more) “classes” of sounds.

**Table 1:** **Perceptible: native speakers report hearing the difference; Unpredictable: the phone cannot be predicted by its phonological context; Lexical: there are many examples of minimal pairs sustaining the contrast**.

Type	Perceptible	Unpredictable	Lexical	Example	First author of relevant study
Phonemic	Yes	Yes	Yes	AmE [b-d]	Whalen; Hacquard
	Yes	Usually	Yes	AmE [I-i]^a^	
	Yes	Mildly	Marginal	ScE [x]^b^	
	Yes	No	Several	Sc [ai-ai:]^c^	
	Yes	No	No	German ich-ach	Hacquard
	Yes	Rarely	Yes	AmE [ 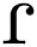 -d]^d^	Boomershine
	No	Rarely	No	AmE [$æ-\tilde{æ}$]^e^	
Allophonic	No	No	No	[p-p*^h^*]	Whalen

(Many examples below are from Scobbie and Stewart-Smith, 2008.)

^a^Typically contrastive, but neutralized in some positions, e.g., seat vs. sit, but see vs. */sI/.

*^b^Only present in a handful of lexical items, e.g., lock vs. loch*.

*^c^Only present in a handful of items, and predicted by syllable structure e.g., side vs.sighed*.

*^d^[d] is typically realized as [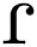] in specific contexts, so they are usually predictable from the context, but there are some cases where they contrast, e.g., rider-writer*.

*^e^Some talkers use more heavily while others do not. E.g., some nasalize in non-nasal environments, or fail to nasalize in nasal environments*.

## The End State: Adults’ Processing of Allophones

It should be noted from the outset that the study of whether listeners’ sensitivity for contrasts that are allophonic is lower than those that are phonemic faces certain methodological roadblocks, which are worthy of discussion here. One way to interpret this hypothesis is the following: Holding the listener and language constant, one would compare a contrast A that is phonemic against a contrast B that is allophonic, to find that A is processed better (discriminated more speedily and accurately; used for tracking phonological patterns; recruited for coding lexical contrasts). Much of the initial literature we review uses this design (e.g., Whalen et al., 1997; McLennan et al., 2003). When using this design, there is an obvious interpretation alternative to phonological status affecting perception: perhaps there is an intrinsic discriminability difference between A and B. A safeguard against this state of affairs is to test two sets of participants, who have different native languages, and hold the contrast constant, an approach that is also common in the literature (e.g., Johnson and Babel, 2010). In this scenario, intrinsic differences in discriminability of contrasts are irrelevant, since only one contrast is used. However, another problem arises, namely that the stimuli must be recorded in some language. If they are recorded in the language where the contrast is allophonic, they may be pronounced less clearly (provided that speakers tend to neutralize such contrasts), which is not desirable. But if they are recorded in the language where they are phonemic, then the test may facilitate the performance of listeners of that language, who will find the stimuli native. The solution that researchers are increasingly adopting is to use a *third* language where the contrast is phonemic, such that the stimuli are equally foreign to both sets of participants. Results from the latter approach actually fit in perfectly with conclusions derived from the two other (e.g., Boomershine et al., 2008), lending further credence to this body of literature.

In brief summary, previous work suggests that adults do not discriminate allophonic alternates as well as phonemes both behaviorally (Whalen et al., 1997; Peperkamp et al., 2003; Boomershine et al., 2008; Shea and Curtin, 2011) and electrophysiologically (Kazanina et al., 2006; Hacquard et al., 2007). Furthermore, adults rate allophones as more similar to each other than phonemes (Johnson and Babel, 2010). Additionally, words differing in sounds that are allophonic prime each other, but words differing in sounds that are phonemic do not (McLennan et al., 2003). These differences in processing come about as the result of native language exposure and thus second language learners have a hard time gaining sensitivity to sounds that are phonemic in the target language even when those same sounds are present allophonically in the learners’ native language (Kondaurova and Francis, 2008).

Thus, overall, perceptual evidence in adults confirms that allophonic and phonemic sounds are not treated similarly. Given that there may be a continuum of allophones to phonemes, as mentioned above, it is worthwhile to evaluate whether this differential behavior arises only for the categorical phonemic/allophonic stages, or also for intermediate cases. This is especially true given recent findings that listeners treat sounds differently based on the reliability of their distributions (Dahan et al., 2008). Specifically, in this study Dahan et al. (2008) examined adults’ perception of tensed and laxed variants of /æ/ in the environment of /k/ and /g/. When /æ/ was consistently tensed before /g/, but not /k/ they found a training effect in the experiment such that listeners, upon hearing e.g., the non-tensed /æ/ came to anticipate the following segment as /k/. Thus, when segments vary allophonically in a reliable way this can lead to differential processing very quickly. This is not the case when the variation is not predictable.

In Table 1, we reclassify adult perceptual studies in terms of the type of contrast that has been examined. There are several studies which explore one of the endpoints (e.g., Whalen et al., 1997 examines a case of clear complementary distribution) and only a few studies which explore points in between. For example, in English [
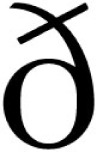
] can never map onto /d/, and therefore they form a phonemic contrast. Whereas [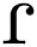] is a possible realization of /d/ in word-medial context, there are also quite a few lexical exceptions where they occur in near overlapping distributions (e.g., rider vs. writer). Thus, the comparison of English adults’ perception of the [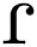] and [d], on the one hand, against [
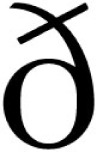
] and [d], on the other, represents the study of an intermediate case of allophony. This was undertaken in Boomershine et al. (2008), who found poorer discrimination of the former than the latter. Results cannot be attributed to the actual acoustic items used, since the same mapped onto different types for a second group of adults, whose native language was Spanish. In Spanish [
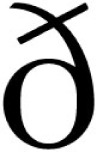
] and [d] are in complementary distribution (classic allophony) and [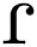] and [
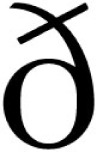
] are mostly in overlapping distribution (classic phonemic). Despite the fact that not all items fell on the extremes of the phonemicness/allophony continuum, perceptual results were the opposite across language groups in all tests but a measure of reaction time. Thus, this work seems to suggest that differences between phonemic and allophonic processing are found even when non-extreme points of the continuum are investigated.

Nonetheless, a different pattern emerges from work using electrophysiology. Hacquard et al. (2007) and Kazanina et al. (2006) both explore cases of complementary distribution, free variation, and overlapping distributions. While there are other effects in these studies (e.g., inventory size), overall, using an oddball paradigm, they find different processing results for complementary distribution (which patterns like overlapping distribution) and free variation (which patterns differently). For example, Kazanina et al. (2006), using Russian and Korean manipulated stimuli, find that while Russian listeners (for whom t/d are phonemic) show a significant mismatch response, Korean listeners (for whom t/d are allophonic) show no such response to the exact same stimuli. Hacquard et al. (2007) also examine whether the amplitude to the mismatch response in an oddball paradigm is related to the phonological status of the sounds in question using synthesized stimuli on vowel tenseness in continental French, Argentinean Spanish, and Puerto Rican Spanish listeners. Tense and lax [e]/[ε] are phonemic in continental French, allophonic in Argentinean Spanish and in free variation in Puerto Rican Spanish and this is reflected in the size of the mismatch responses. Furthermore, Argentinean listeners seem to discriminate the allophonic differences as well as they discriminate the phonemic ones based on the size of the mismatch response, but Puerto Rican listeners seem to discriminate the allophonic/free variation contrast more poorly than a phonemic contrast ([e]/[a]). Thus, theoretical descriptions and psycholinguistic evidence suggests that allophones and phonemes are different and that typology of the allophones may also be a factor in processing at least at some level. The next section assesses when these differences in processing come about over the course of development.

## Infants’ Processing of Allophones

As mentioned above, a considerable body of literature suggests that perception of non-native (absent) sounds declines, whereas perception of native phonemes improves toward the end of the first year of life (Polka et al., 2001; Kuhl et al., 2006; Narayan, 2006). This improvement likely relates to the much richer and more abundant cues for the former: The child will accumulate more passive, phonetic exposure to the former; she may attempt these sounds; she may learn some words that have them, and so forth. The first question we would like to answer is when listeners become less sensitive to allophonic distinctions and more sensitive to phonemic ones. We review evidence from discrimination, phonotactic learning, phonotactic processing, and word learning suggesting that infants are sensitive to phonological status.

English-learning 2-month-olds discriminate allophonic variants (e.g., /t/ in “night rate” versus “nitrate”; Hohne and Jusczyk, 1994) showing an initial sensitivity to sounds that will eventually be treated as allophones later in life. Recent work suggests that, while young infants are sensitive to sounds that are allophones in their ambient language, this sensitivity declines with maturation and language-specialization. Specifically, Seidl et al. (2009) briefly familiarized English- and Quebec French-learning infants with a pattern that depended upon vowel nasality. Note that as mentioned earlier vowel nasality is phonemic in French, but allophonic in English. Infants in this study heard syllables in which nasal vowels were followed by fricatives, but oral vowels were followed by stops. Then they were tested on their ability to generalize this pattern to new syllables. English-learning 4-month-old infants were able to learn this novel phonotactic dependency involving vocalic allophones and behaved like French-learning 11-month-old infants, for whom nasality is phonemic. However, by 11 months of age English-learners were no longer able to encode this abstract phonotactic regularity and showed no evidence of learning. It should be noted that these older infants are not completely impervious to allophones, since they use them to extract words from running speech at 10.5 months (Jusczyk et al., 1999). Rather, these results suggest that the same exact sounds no longer function in the same manner across languages which use them as phonemes versus allophones.

It might be suggested that some of the contrasts that have been studied as allophones could be more perceptually difficult than ones that have been explored as phonemes. Specifically, allophonic alternates may simply be more difficult to discriminate because they represent subtle changes. For example, Pegg and Werker (1997) found that two phones that map onto different phonemes /t/ and /d/, but are extremely similar, are not discriminable by one-year-olds. In their study, they measured sensitivity to the word-initial realization of /d/ against the post-/s/ realization of /t/, which differ very subtly. However, an important point is that simple acoustic distance between the tokens used in any given test cannot explain developmental changes in allophonic sensitivity, since this sensitivity changes with age and language exposure. Even in the Pegg and Werker (1997) study, 6-month-old were, in fact, able to distinguish the very similar surface realizations of /t/ and /d/. Similarly, Dietrich et al. (2007) and Seidl et al. (2009) show that attention to the same contrast declines in languages for which they are allophonic, but not in languages in which they are phonemic. For example, Dietrich et al. (2007) show that 18-month-old Dutch-, but not English-learning toddlers interpret vowel length as lexically contrastive. Thus, it appears that while sensitivity to allophonic sounds initially exists in infancy, it appears to decline by 11 months of age (Seidl et al., 2009) as infants converge on the native phonemic contrasts present in their input language and come to ignore the non-native ones which are not present in their input language (Werker and Tees, 1984).

It is worthy of note that most of the studies cited above have been conducted on English-learning infants (albeit with two exceptions, Dietrich et al., 2007; Seidl et al., 2009). If we are to draw any clear conclusions concerning the time course of allophonic sensitivity, we will need to expand this work cross-linguistically, since it may be that the time course is different across languages and may also be impacted by the kind of sound distinction explored. Unfortunately, such single language studies only allow for certain allophonic sounds to be tested, and confound potential differences in discriminability with phonological status.

Also worthy of mention is that many allophonic alternates in the studies mentioned above are predictable from the phonological context. For example, the aspiration of /t/ studied in Hohne and Jusczyk (1994) represents a clear case of complementary distribution or classic allophony. Exceptions to this are the cases of vowel nasalization utilized in Seidl et al. (2009) and the case of vowel length in Dietrich et al. (2007). Specifically, although vowels are nasalized before tautosyllabic nasal consonants in English, they are also often nasalized in other locations (e.g., within a word with another nasalized vowel), so complementary distribution does not entirely hold. Thus, although there are cases where nasalization of vowels is completely predictable on phonotactic grounds (before nasal Cs in the same syllable), we also see nasalization in other locations for coarticulatory reasons. To add more complexity to this picture, variation in nasalization has been reported across American English dialects, such that nasalization could become a sociolinguistically relevant feature (e.g., a marker of African American Vernacular English), more than a phonotactically relevant one. Similarly, in Dutch we see a case where vowel length is difficult to classify using our classic definition of allophony. Although there are minimal pairs with vowel length in Dutch, the presence if minimal pairs occurs unevenly across the inventory. For example, /
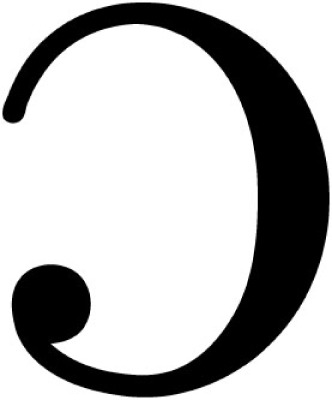
/ has long and short minimal pairs that differ mostly in length (although there are slight vowel quality differences). All other vowels that have been described as contrastive in length show considerable changes of vowel quality with the addition of length, much as we see in English tense-lax pairs. Certainly, the infant literature is not rich enough to conclude that there are no differences among the different degrees of allophony. Nonetheless, current research suggests that in infants, as in adults, even degrees of contrastiveness may make a difference, with more allophonic pairs being processed less well than more phonemic pairs. Across all studies, however, it appears that younger infants attend to salient distinctions more than older infants when the distinctions are allophonic in the target language.

## Mechanisms for Learning Allophones

Young toddlers treat allophones as distinct from phonemes. Further, some of the evidence reviewed suggests that they come to do so within the first year of life. How does such a young toddler come to treat allophones as distinct given that they clearly vary from language to language? Or more specifically, how do they come to attend less to allophonic sound pairs and attend more to phonemic sound pairs? There are several possible answers. Below, we describe computational models and laboratory studies documenting the ways by which allophonic treatment could come to be distinct from phonemic treatment.

### Phonetic mechanisms

One possibility for learning the difference between allophones and phonemes is that phonological status may be partially coded in the acoustic signal. Specifically, it may be that allophonic alternates are less distant from each other than phonemic ones; this difference could ensue because speakers produce them less clearly since their listeners pay little attention to them and thus communication is not compromised by their lack of distinctiveness; or simply because speakers themselves do not hear the difference very clearly, and thus never hyperarticulate these sounds. Such a strategy appears to be a cheap and sensible one, since infants are extremely sensitive to the acoustic properties of phonemes in their input (Maye et al., 2002; McMurray and Aslin, 2005; Cristia et al., 2011).

Corpus studies confirm that phonological status is, indeed, coded in the acoustic signal. Yuan and Liberman (2011) measured the Mel-frequency cepstral coefficients (MFCCs) of nasal and oral vowels in three languages (Mandarin, Portuguese, English) and after training used a classifier to sort the vowels into either nasal or oral classes. Results revealed that classification was easier for Portuguese, a language with phonemic nasality, than in either English or Mandarin, languages in which nasality is allophonic. Thus, these data support the idea that there may be acoustic cues to the classification of either phoneme or allophone, such that phonemes are more distinct and hence more easily classified using MFCCs.

Similar findings may obtain in infant-directed speech. In recent work, Cristia et al. (2010) measured two different phonemic and allophonic contrasts in infant- and adult-directed speech in corpora of Quebec French and American English. Specifically, they explored tenseness which is phonemic in English (“bit” vs “beet”), but allophonic in Quebec French: In Quebec French tense vowels are laxed in closed syllables. They also explored vowel nasality which is phonemic in Quebec French (“mode” vs “monde”), but allophonic in English: In English vowels are nasalized before tautosyllabic nasal consonants. After collecting corpora of both tense and lax, and nasal and oral vowel pairs in each language in phonologically controlled environments and in both infant- and adult-directed registers, they conducted acoustic measures of Euclidean distance between vowel-specific alternates (nasal/oral, tense/lax) using traditional acoustic measures of tenseness and nasality. Results revealed that in terms of acoustic distance the tense/lax pairs of vowels were closer in the allophonic language than in the phonemic one regardless of the specific vowels explored. Nasality, on the other hand, was equally marked in both the phonemic (French) and the allophonic (English) language. While it may be the case that this unevenness was found because nasality is simply more difficult to measure acoustically than tenseness, if we take this data at surface value it appears that the phonemic vs allophonic distinction is better marked in some areas of acoustic space than others.

Although some information on phonemic status is clearly present in the signal, corpora studies cannot reveal whether the infant learner actually uses this acoustic information about the “closeness” of sounds in her phonological processing. Further work is necessary to answer this question.

While the argument of phonetic similarity is convincing for some cases of allophony, it is unlikely that it could explain perceptual desensitization for all sounds that adults treat as allophones. An intuitive case in point is that of /t/ allophones in English varieties, which can sometimes (albeit rarely) be realized as glottal stops. There is *a priori* no reason to imagine that [t] and [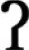] are similar; and certainly not more similar than [k] and [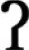] (that is, if [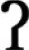] has to be the allophone of some sound, phonetically it is much closer to /k/ than /t/). In view of such arguments, researchers have also explored other mechanisms, to which we turn.

### Distributional mechanisms

An additional possibility is that infants use distributional cues, meaning the context in which a phone occurs, to discern between allophones and phonemes. For example, in English aspirated /t/ and unaspirated /t/ do not occur in the same location, so complementary distribution can effectively be used as a key to the allophonic categorization of sounds in classical phonemic versus allophonic cases. This strategy seems a sensible one since evidence suggests that young babies may be sensitive to distributions of syllables (e.g., Saffran et al., 1996) and sounds (e.g., Chambers et al., 2003; Seidl and Buckley, 2005; Cristià and Seidl, 2008; Seidl et al., 2009).

These distributional mechanisms have received support from a recent artificial grammar learning study. White et al. (2008) explored the effects on infants’ perception of exposure to an artificial grammar that could be described as having morpho-phonologically conditioned allophony. Specifically, they familiarized 8- and 12-month-old infants with a grammar containing “determiners” followed by “content” words in which voicing of the initial C of the content word alternated as a function of the voicing of the final segment of the function word, but only with consonants of certain manners. Note that this represents a slightly different sort of allophony than the sorts discussed above, since the “complementary distribution” did not apply within the “content” words, but it was nonetheless still predictable. While 8-month-olds were able to learn these patterns, only 12-month-olds seemed to have grouped the alternate variants into a single functional category.

In addition, computational modeling also provides some support to the complementary distribution strategy. Peperkamp et al. (2006) investigated the performance of a model that categorized sounds in complementary distribution as allophones, and sounds with overlapping distributions as phonemes. This algorithm was tested on both an artificial language as well as a simplified corpus of phonetically transcribed French. While the algorithm did well in correctly tagging allophones in the artificial grammar, its performance was more error-prone in the French language corpus. Specifically, it over-generated, generating allophonic alternates that were not actually present in French. Errors of this kind were reduced to a certain extent if phonetic proximity was also taken into account.

Peperkamp et al. (2006) also suggest that these errors occur because of the presence of many near-complementary distributions, as mentioned above. Specifically, it is the cases that exist along the continuum between allophones and phonemes, but not at the edges of this continuum, which are difficult for the algorithm to correctly classify. These may be problematic to all learning algorithms of this kind (and, though evidence does not yet support this, to infants as well!). However, since near-complementary distributions are present in natural languages and there is no clear cut-off point along the continuum that has been found, it may be that until we discover how humans process these cases along the continuum we will not be able to create algorithms to do so.

In concert, experimental and modeling results support the contribution of distributional information for learning of certain cases of allophony. They also underline that distributional information alone is not sufficient, but must be packaged together with acoustic similarity. This is not a limitation, as it is likely that multiple mechanisms work in concert for the discovery of phonological status.

### Lexical mechanisms

The most informed, or high-level, source of information for phonological status involves semantic knowledge. Jakobson (1966) proposed that children use semantic cues, essentially using minimal pairs to discern which phonemes are crucial to the input language and which are not. Thus, a child might hear palatalization in English before [j,i,e]. Thus, she will hear at least two different alternate pronunciations of the word *hit*. Specifically, she will hear *hi[c] you* for “hit you,” but also hear *hi[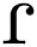] him* for “hit him.” Both of the utterances will be uttered on occasions where hitting takes place. On a lexical account, the child would decide that these two instances of *hit* must map to the same underlying structure, /hɪt/. In addition, the child will be at the same time learning which sounds are phonemes by calculating minimal pairs. Thus, the child will learn that /s/ and /h/ are distinct phonemes of English because *sit* and *hit* map onto different semantic representations. Indeed, Yeung and Werker (2009) experimentally demonstrated that infants regain attention to a non-native contrast after seeing the members of the contrast paired with different visual referents. These two processes, one of semantic overlap and one of semantic distinction, may occur together and drive children’s developing phonological representations. In a certain sense we can rule out the strong version of this hypothesis as the sole method of learning given that infants at 11 months in Seidl et al. (2009) treated allophones as distinct from phonemes. Specifically, because infants at 11 months (and likely even older: Dietrich et al., 2007) do not have many minimal pairs (Caselli et al., 1995) it seems unlikely that they can use lexical cues as the sole driving factor in their phonological category learning.

Thus, the old-style lexical hypothesis seems not to hold much promise. However, a new version of lexical bootstrapping has emerged in recent years. This work is based on the finding that minimal pairs can be insufficient for the learner to maintain a phonological distinction, and that near-minimal pairs are more useful for deciding on phonemic dimensions. Thiessen (2007) documents that 14-month-olds presented with a perfect minimal pair based on stop voicing (such as taw-daw) fail to discriminate two syllables differing along that feature, whereas toddlers exposed to near-minimal pairs (such as tawbow and dawgoo) have an easier time. Swingley (2009) goes further to propose that infants could use commonalities in the pronunciation across otherwise completely different forms (such as the first vowel in *yellow* and *better*, something one could describe as “maximal pairs”) to extract sound categories, and argues that this new type of lexical bootstrapping could make a considerable contribution to infants’ phonological acquisition. Swingley and collaborators have recently bolstered this case by reporting that 6-month-olds have referential knowledge of several words (Bergelson and Swingley, 2012), such that their lexicon could be slightly larger than previously thought (Tincoff and Jusczyk, 1999). Moreover, corpora analyses showed that infant-directed speech offers few true minimal pairs, but rich maximal pairs structure, which an informed machine learner can profit from to learn about the phonemes of her input language (Swingley, 2009). We expect that a similar training study with infants is underway, which would constitute the final pre-requisite for this view of lexical bootstrapping. These new versions of lexical bootstrapping assume that infants can use semantic information to pull apart phonological categories. It should follow, then, that in the absence of such separating forces, infants could collapse allophonic sounds. More specifically, if maximal pairs are necessary to establish sounds as contrastive then the absence of such pairs may aid in establishing similarities between structures and assigning phonological alternations/allophonic relationships. Thus, this same mechanism might help the toddler establish that the *I* in *I’d* and *I’m* map onto the same representation. To our knowledge, the latter argument has not been made by proponents of lexical bootstrapping of phonology, but we foresee such a theoretical development within that promising line of work.

A second strain of models of phonological acquisition does not assume rich semantic representations to separate the sounds, but proposes that infants hold a pseudo-lexicon, a dictionary of frequently encountered wordforms (Martin et al., in press). In this proposal, wordform minimal pairs are used to detect allophones, such that if the child’s lexicon contains two (long) sequences of sounds that are identical except for one sound, then the two sounds that differ across the two stored sequences should be considered allophones of the same phoneme. Using such an algorithm, phones could be classified as allophones and phonemes with a much greater accuracy than with other algorithms using only distributional information, or a combination of distributional information and acoustics (detailed in the Distributional mechanisms section). A pre-requisite for this type of lexical bootstrapping is that the child has a proto-lexicon, a wordform repository. Recent experimental work corroborates this: 11-month-olds showed no preference between sequences of phones that were frequent in their input, but which did not form real words, and actual real words (Ngon et al., in press). In contrast, they do prefer frequent words over infrequent words (Hallé and de Boysson Bardies, 1994), and frequent sequences over infrequent wordforms, even when phonotactics had been controlled for (Ngon et al., in press). The next step in the exploration of this potential explanation for phonological acquisition involves showing that infants use minimal wordform pairs to *collapse* across the distinction, rather than separate it. If this prediction holds, it would demonstrate that minimal wordform pairs and true, lexical minimal pairs do not operate in the same fashion at all.

A variant of the latter hypothesis could be proposed where long-term storage and the assumption of different mechanisms governing wordform and lexical minimal pairs are unnecessary. It is well known that infant-directed speech abounds in repetition, with a much greater narrowness of focus than adult-directed speech (McRoberts et al., 2009). In other words, it appears that infant-directed speech exaggerates “burstiness” (Baayen, 2001), the tendency for lexical items to recur within the same conversational interaction, in a way that could influence phonological acquisition (Skoruppa et al., 2012). A smart learner may be able to use variation across two wordforms experienced in close succession to derive probabilities of non-contrastiveness. For example, if the child hears “dad,” “da[d]y,” “da[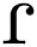]y” in the same conversational interaction, she may be able to store that [d] and [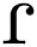] could be variants of the same phoneme. The latter extension has not yet been espoused by modelers, but we expect it may be just around the corner. The predictions from this hypothesis could also be easily tested using an artificial grammar design.

Whereas the combination of acoustics and distributional cues seemed to gain the learner-model quite a bit, some work suggests that a learner-model combining distributional and lexical mechanisms, or all three together, may only be subtly improved (Boruta, 2011). It is of theoretical and empirical interest to thoroughly investigate the effects and interactions emerging from the integration of all 3 types of mechanisms in the future.

## Implications

Collectively, this work suggests that multiple mechanisms, likely including the computation of complementary distribution and the calculation of phonetic similarity, operate in concert to guide infants toward their functional interpretation of sounds that are present in the input, yet not contrastive. This review also bears on the more general question of how infants cope with phonetic variability that is not lexically meaningful such as variation between talkers’ voices and accents. Interestingly, infants become resilient to talker and accent changes also toward the end of the first year of life (Houston and Jusczyk, 2000; Schmale et al., 2010). Future work should investigate whether this similarity is merely superficial, or whether it is indicative of a perceptual reorganization allowing toddlers to recognize wordforms in the presence of lexically irrelevant variation. To answer this question, research should focus on how infants cope with deviations from canonical productions and how predictable those productions are. Moreover, the question of allophones is a categorical one, but many sources of variance are gradient and future work should explore whether these different kinds of variation are more or less learnable since it may be that gradient changes to the acoustic character of a sound are more variable.

A second consideration relates to the nuances in the concepts of phonemes and allophones laid out above, and predictions that can be stated on their learnability. Recent artificial grammar learning work suggests that infants tend to attend more to regular, neither entirely predictable nor entirely unpredictable, patterns (Gerken et al., 2011). In the domain of allophonic learning this might translate to different attention being allotted to patterns that are halfway between allophones and phonemes because of their very irregularity, a matter that could be investigated by assessing infants’ acquisition of different types of phonemes/allophones. Additionally, one could imagine that for infants the areas of the grammar in which the irregularity resides may be very important. For example, if the irregularity is lexically or morphologically based the language learning infant may not be immediately aware of it, and so would initially treat the pattern as if it were regular.

Additionally, differential processing of allophones and phonemes could inform translational research. For example, some work suggests that inappropriate learning of sounds in terms of these sound classes (e.g., perceiving equally well different phonemes and different allophonic alternates) correlates with reading ability and differs between normally developing and dyslexic children (Serniclaes et al., 2004). If we can pinpoint these differences in early development it may be possible to intervene while these infants are still at a very plastic stage of development. Thus, longitudinal studies exploring allophonic and phonemic processing may well contribute to early intervention at some point in the future.

Although we have steered clear of production in this review, it is certainly the case that accurately representing sounds as mapping onto distinct phonemes or the same phoneme should relate to production, since the target phonology for production will require the child to use the underlying sound in different ways in different environments. All signs indicate that this is a process that occurs quite early in development (Fikkert and Freitas, 2006). Still it is unclear to what degree the continuum between allophones and phonemes relates to production of those categories. We leave that question for a future review, but mention here that it is crucial to unite these two processes within the infant in order to truly understand the course of infant development.

It remains unclear how infants might make use of “phonetic similarity” in discovering allophones and distinguishing them from phonemes. For example, all vowels are more similar when compared with consonants, yet even young infants do not appear to have difficulty in distinguishing one vowel from another. It may be crucial to discern how acoustic similarity is judged vis-a-vis the infant. It is possible that lexical factors may also play a role in infant learning of phonological categories in a greater way than has been shown in learning models (Swingley, 2009).

Finally, it is clear that allophones may be relevant not just to phonological learning, but also to syntactic learning since allophonic alternates may mark phrasal edges (Selkirk, 1984; Nespor and Vogel, 1986; Seidl, 2000) and this marking may help infants to learn their syntactic structure if they are attentive to these edges (Nespor et al., 1996; Christophe et al., 1998). For example, if there is strengthening of contact at domain edges (Keating et al., 2003) or specific phonological processes at domain edges as mentioned above, e.g., a greater degree of aspiration or longer linguo-palatal contact the higher up you go in the prosodic hierarchy, then if infants are aware of the prosodic cues that they use for syntactic bootstrapping, this knowledge should inform or a least interact with their acquisition of the knowledge of allophones.

## Conflict of Interest Statement

The authors declare that the research was conducted in the absence of any commercial or financial relationships that could be construed as a potential conflict of interest.

## Footnotes

^1^“Allophone” is used somewhat variably across papers. For example, some use the word to denote the more marginal pronunciations of a sound (e.g., if vowels are nasalized before nasal vowels and are oral elsewhere, then some would call the nasal alternate an allophone and the oral one a phoneme, Peperkamp et al., 2006). In more traditional phonological terms, all sounds are allophones, surface representations that map onto some phoneme (abstract representation). Following this definition, for example, one should state that in English oral and nasal [i] are allophones of the same phoneme, whereas oral [i] and oral [e] are allophones of different phonemes. We adopt the latter definition, except that for ease of reading we will refer to cases like the previous one (allophones that map onto the *same* phoneme) as “allophones,” and to the latter (allophones that map onto *different* phonemes) as “phonemes.”

^2^One anonymous reviewer points out that this problem only exists in the case that the speech stream is segmented, since only segmentation into syllables would lead to the conclusion that the allophone of /t/ that occurs in “st” clusters does not group with the phonetically similar [d], but rather with the phonetically dissimilar [t]. It is unclear when exactly infants segment into syllables. Some data point to syllables being the basic unit of analysis even for newborns, allowing the discrimination of /atspa-apsta/ but not that of /tsp-pst/ (Bertoncini et al., 1988); while other data suggests a protracted development, as infants do not use the syllable-determined allophones of /r/ in “night rate” versus “nitrate” to segment these words from running speech until about 10 months of age. Thus this is certainly a question that warrants further exploration.

^3^This sort of “active” contrast is eminently common in sign languages, which have very few clear minimal pairs. Thus while a few clear minimal pairs exist e.g., in the domain of handshape (Brentari, 1998), most are cases of near-minimal pairs or cases where a contrast exists in one area of the lexicon, but looks distinct in another area of the lexicon (Brentari and Eccarius, 2012). For example, according to Diane Brentari (p.c.) The ASL sign THOUSAND was originally borrowed from the initialized French Sign Language sign MILLE and had a 3-finger “M” handshape. During the process of nativization the “M” (3 fingers) became “B” (all 4 fingers). That is the more marked 3 fingers became the less marked 4 fingers handshape. However, in this location with this movement there are no minimal pairs with either of these handshapes.
